# Appreciation should be EA-SI—demystifying the definition and operationalization of experienced appreciation at work by developing a new construct

**DOI:** 10.3389/fpsyg.2025.1445533

**Published:** 2025-09-01

**Authors:** Maximilian Resch, Henrik Bellhäuser

**Affiliations:** Psychology in Education, Institute of Psychology, Johannes Gutenberg University Mainz, Mainz, Germany

**Keywords:** EA-SI, EA-SI work scale, stress as offense to self, occupational health, wellbeing

## Abstract

In this article, we developed the new construct, Experienced Appreciation in Social Interactions (EA-SI), to reduce the inconsistency in defining and measuring experienced appreciation at work. The integrative theoretical model is based on the well-validated *Stress as Offense to Self*-theory. To operationalize the construct, we validated the EA-SI Work Scale in two independent German samples of employees. Colleagues and supervisors were investigated as potential sources of experienced appreciation. Study One included *N* = 231 participants. Study Two encompassed *N* = 391. In both studies, we applied a cross-sectional field-study design based on self-reported surveys. Using exploratory and confirmatory factor analyses, the construct EA-SI turned out to be unidimensional. The Pearson product-moment correlations showed that the more employees felt appreciated, the higher their self-esteem and the lower their stress perception. The premises of the theoretical foundation were replicable. Higher experienced appreciation was related to more work satisfaction, life satisfaction, and work engagement, as well as lower emotional exhaustion. These relations were true for both groups of appreciators. When tested in hierarchical regressions, EA-SI added incremental prediction beyond the influence of social support in most of the analyses. The instrument's internal consistency and retest reliability were good to excellent. The results indicated the EA-SI Work Scale to be content, construct, and criterion valid. Based on these findings, the strengths and limitations of the article and possible implications for future research and practical use are discussed.

## 1 Introduction

Relationships should be based on the four principles: respect, understanding, acceptance, and appreciation to foster joy, fulfillment, and harmony in our everyday interactions. At least, that is what Arun Gandhi postulated about non-violent social interaction (Gandhi, 2017). In line with Gandhi's evaluation, occupational health psychology—acknowledging the work context as one of the most important origins of individuals' identity and self-esteem (Ashforth and Schinoff, 2016)—also focuses on investigating appreciation.

Feeling appreciated at work relates to increased motivation and satisfaction (Kottwitz et al., 2019; Stocker et al., 2010), higher serenity at the end of the workday (Stocker et al., 2014), reduced stress and anxiety (Teo et al., 2021), and increased positive affect toward the organization (Pfister et al., 2020). Appreciation buffers the negative relation between interruptions at work and wellbeing (Stocker et al., 2019) and is associated with reduced turnover intention (Apostel et al., 2018) and burnout (Teo et al., 2021).

Despite its importance, appreciation seems to be a resource of limited availability. On average, employees reported less than one single appreciative interaction a day (Stocker et al., 2014). Moreover, the construct “appreciation” is inconsistently defined and operationalized in scientific literature. For research to move forward effectively and for practical use—such as enabling employees and supervisors to foster appreciative experiences purposefully—the construct should be defined in a congruent, unambiguous way to attain comparable scientific findings.

In contrast, research often entangles appreciation with other constructs such as social support (Sundin et al., 2007), reward (Bregenzer et al., 2022), gratitude (Tang et al., 2021), respect (van Quaquebeke and Eckloff, 2010), or social recognition (Schneickert et al., 2019). In distinction from this approach, Semmer et al. (2019) understand appreciation as a unique construct that should not be synonymously entangled with other constructs.

Even when defined as an autonomous construct, there is no consensus regarding the theoretical definition and framework of appreciation. Most definitions focus solely on conditional appreciation. Unconditional expressions of appreciation—such as valuing one another as human beings regardless of performance or achievements—are oftentimes not integrated explicitly. In line with the incongruency in defining appreciation, its assumed factor structure varies from five-factorial (White, 2016) over four-factorial (Döring-Katerkamp and Rohrmeier, 2016) to single-factor solutions (Bakker et al., 2007; Carstensen et al., 2021; Semmer et al., 2019).

Please note that for a more detailed illustration of different understandings and operationalizations of appreciation, Resch et al. (2025) could be consulted. Given the described research gap and following the findings by Semmer et al. (2019), pointing toward appreciation as an autonomous construct, the current article aims to answer the following research questions:

**Q1:** How can the different understandings of appreciation be combined in one integrative construct definition that values conditional and unconditional aspects?
**Q2:** What is the factor structure of this new construct?
**Q3:** Can experienced appreciation be reliably and validly measured using the new construct?

Previous studies have reported significant relations between appreciation and employees' wellbeing, satisfaction, and motivation. However, some of these studies were based on samples with little heterogeneity, challenging the generalizability of the results. For example, the data was limited to students (Carstensen et al., 2021), nurses (Muntz and Dormann, 2020), or soldiers (Stocker et al., 2010) only. In addition, most articles solely considered work-related outcomes, while potential spillover effects from work to other contexts of employee life (Liu et al., 2020) were ignored. Hence, we propose a fourth and fifth question:

**Q4:** Is experienced appreciation solely associated with work-related measures, or are there spillover effects on other aspects of employees' lives?
**Q5:** Are the expected relations replicable in branch-heterogeneous samples across different professions?

Finally, it should not be neglected that there are intersections between the constructs “social support” and “appreciation” (Kurtessis et al., 2017). To test the assumption by Semmer et al. (2019) that appreciation is a construct that predicts employees' wellbeing above social support rather than being a facet of the same, we propose a sixth research question:

**Q6:** Is appreciation theoretically and statistically distinguishable from social support?

Following these research questions, this article aims to develop and validate a new construct—Experienced Appreciation in Social Interactions (EA-SI)—that combines different understandings of appreciation in one integrative model. To do so, we theoretically derived the construct from literature and subsequently investigated it in a multi-study field design with two branch-heterogeneous samples across Germany. In both studies, we focused on colleagues and supervisors as possible origins of appreciation (e.g., Stocker et al., 2014).

To counter the incoherence in defining and measuring appreciation at work, in the following article, we will (1) derive a new construct based on different understandings of appreciation and (2) unravel the Stress as Offense to Self-theory (SOS; Semmer et al., 2019) as the theoretical foundation of EA-SI. We will then (3) develop the EA-SI Work Scale to operationalize this new construct. In Study One, we will (4) statistically revise the instrument, and (5) exploratively investigate the factor structure of EA-SI, as well as (6) test the reliability and criterion validity of the EA-SI Work Scale.

In Study Two, we will (9) test the assumed factor structure of EA-SI and (10) replicate the theoretical premises of the SOS theory in an independent sample. We will (11) test for convergent and discriminant construct validity and (12) investigate the incremental validity of EA-SI in predicting employee wellbeing, engagement, and satisfaction above social support. Finally, the findings of both studies, their strengths and limitations, and implications for research and organizations will be discussed. Please note that the findings and implications of the current article inspired the development of a short scale to measure EA-SI, extending the understanding and validation of the construct (Resch et al., 2025).

## 2 Theoretical framework

### 2.1 Theoretical considerations in deriving EA-SI

Referring to Merton (1968), implying that theoretical frameworks should be distinguished regarding different levels of scope and depth, we derived EA-SI considering not solely grand theory but also “middle-range theory,” and applied theory. Grand theory should strive to explain “the uniformities of human behavior […] refer[ring] to logically interconnected sets of propositions from which empirical uniformities can be derived” (Merton, 1968, p. 39). On this overarching level, EA-SI acknowledges the grand theoretical belief that human beings are social entities, sharing the uniform need to (socially) connect to each other (Baumeister and Leary, 2017). Following this need to connect, human beings must communicate with each other, whether actively intending to do so or not (Watzlawick et al., 2016). This ongoing communication involves either understanding or misunderstanding, depending on the encoding and decoding process of messages sent and received (Schulz von Thun et al., 2014). Coherently, these interactions can be characterized by the experience of feeling more or less appreciated. These theoretical considerations are understood as basic axioms of human interaction on the most generic level and are not further tested in this article.

On the level of “middle-range theory”—explaining specific phenomena on a more small-scaled level (Merton, 1968)—EA-SI is based on the assumption that interpersonal interactions should be characterized by an appreciative and benevolent attitude toward each other, in order to maintain and protect a positive self-esteem. Hence, we built EA-SI upon the SOS theory, putting the human self-esteem and its relation to appreciation in the center of the theoretical framework. The SOS theory will be outlined in detail in Section 2.3 and tested statistically in both studies.

Since there are several mutually inconsistent understandings of appreciation on the level of applied theory—offering the most small-scaled, hypotheses-based theoretical considerations benefitting practical application—we decided to counter this incongruency by defining EA-SI as an integrative construct that combines a variety of different signals and/or actions that are believed to foster the feeling of being appreciated. This is why the construct's definition is circulating around a diversified list of explicit manifestations of appreciation rather than equating a definition on a more generic level. In coherence with the described theoretical incongruency, we did not propose the expectation of a specific factorial structure but investigated the dimensionality of EA-SI exploratively in Study One before replicating it in Study Two.

### 2.2 Defining experienced appreciation in social interactions

Following the word appreciation to its linguistic roots, the Latin word “appretiare” (OED Online, 2013) means rating something or someone. In a more contemporary sense, the verb “to appreciate” means to see, cheer, and acknowledge someone's value (Schäfer, 2021).

When examining how the scientific literature addresses appreciation, it is apparent that there are many constructs associated with the same word. Appreciation could describe the awe and wonder one feels when thinking about oneself and one's life (Adler, 2002; Fagley, 2016), as well as the positive acknowledgment of one's own body (Avalos et al., 2005) or the positive evaluation one receives from others (Pfister et al., 2020; Semmer et al., 2019; Stocker et al., 2010).

This article focuses on the appreciation experienced when interacting with others. EA-SI differentiates between the person who feels appreciated (*appreciation receiver*) and the one who sends appreciative signals (*appreciator*). Since the model integrates fundamental theoretical considerations regarding interpersonal communication (Schulz von Thun et al., 2014; Watzlawick et al., 2016), how the receiver decodes and interprets a sent signal should substantially determine whether or not they feel appreciated. Hence, EA-SI focuses primarily on the receiver's perception and sensations rather than the appreciator's intentions.

The appreciation receiver can experience appreciation directly and verbally by the appreciator as well as indirectly and non-verbally (Stocker et al., 2014). Such interactions can occur publicly in front of others or in more private one-on-one settings (Adair, 2009). For example, finding a small note with the words “Thank you!” written on it (indirect/verbal/one-on-one) is also a manifestation of EA-SI as hearing the same words spoken out aloud during a team meeting (direct/verbal/public).

The following definition of EA-SI combines different understandings of appreciation and specific actions and/or messages that are believed to transport appreciative signals in a single construct. EA-SI means feeling conditionally acknowledged for achievements and competencies (Stocker et al., 2014). It means that the appreciation receiver has the feeling that the appreciator listens to them and is authentically interested in their contributions (e.g., Stocker et al., 2019; Yukl et al., 2002). The more the appreciation receiver feels respected (Apostel et al., 2018), valued (Kuoppala et al., 2008), and trusted (Semmer et al., 2007), the higher EA-SI should be. EA-SI is manifest when the appreciation receiver experiences an investment of time and resources to ensure their wellbeing, as well as adequate material and non-material recognition (e.g., Semmer et al., 2019), and opportunities for personal and professional development (Siegrist, 2016). EA-SI encompasses the feeling of being unconditionally valued for one's personality or habits as a human being, regardless of any terms. Integrating fundamental considerations of client-centered (Rogers, 1991) and non-violent communication (Rosenberg, 2015), EA-SI means getting the opportunity to socially and emotionally bond with the appreciator, as well as feeling treated in a non-violent way. Following the premise by Mettler-von Meibom (2007) that the way individuals communicate with each other should be more important in shaping their relationship than the actual message sent, the described actions combined in EA-SI reflect an underlying loving and benevolent attitude of the appreciator experienced by the appreciation receiver.

Going beyond social support—defined as helping behavior toward others (when times are challenging)—appreciation means recognizing one's counterpart not solely in demanding situations but throughout the ups and downs of their work (Pfister et al., 2020). EA-SI includes acknowledging the appreciation receiver in times of success as well as strengthening them when times are tough (Resch et al., 2025). For a more detailed differentiation between appreciation and social support, Semmer et al. (2019) can be consulted.

In the following deduction of the hypotheses, different studies will be cited that relate appreciation to specific outcome measures. Please note that the results are based on incongruent construct definitions and operationalizations. Nonetheless, these findings are invaluable to deriving the relevance of experienced appreciation for scientific and occupational applications and to pointing out the necessity of developing the new, integrative construct EA-SI. For a more detailed overview of various understandings of appreciation, see also Resch et al. (2025).

### 2.3 Theoretical integration of EA-SI

We built the EA-SI model upon the assumptions of the well-established SOS theory (Semmer et al., 2007, 2019). To develop a solid theoretical foundation, we will comprehensively discuss other established theories and justify our decision for the SOS theory.

#### 2.3.1 The SOS theory as theoretical framework for EA-SI

In occupational health, various theories exist to explain employee wellbeing, motivation, and satisfaction. Hence, the question arises upon which of these theories EA-SI should be built on. Besides the SOS theory, two other well-known theories are Bakker and Demerouti Job Demands-Resources theory (2014; JDR) and the Conservation of Resources theory (Hobfoll et al., 2016; COR).

The JD-R differentiates between resources and demands at work. Higher resources are associated with increased motivation, while higher demands lead to increased strain at work. Both pathways are believed to influence each other intersectionally. Ultimately, employee motivation and strain result in organizational outcomes such as absenteeism (Clausen et al., 2012) or job performance (Bakker et al., 2007). The COR focuses on the maintenance and protection of resources. It combines personal, social, and structural resources by assuming that the loss of these resources results in the sensation of stress. In contrast, conserving these resources should be a protective strategy against stress. Consequently, the loss of resources—weighing heavier than gaining new resources—should result in adverse (organizational) outcomes (Hobfoll et al., 2016).

Thus, the JD-R and COR define different resources and demands that can intensify or buffer each other within complex interplays. While the JD-R distinguishes between demands and resources, the COR theory distinguishes between three distinct categories of resources. In contrast, the SOS theory puts the individual's self-esteem in the center of attention. The theory proposes that it is a basic human need to maintain and protect a positive self-esteem. The SOS theory defines specific boosts and threats that are directly related to the self (Semmer et al., 2019). Other than resources that can be buffered crosswise, the presence of a threat to the self represents a severe attack on the underlying human need, resulting in a stress reaction.

This deep-reaching focus on the human self-esteem is where the focal point of the SOS theory differs from other theories. Experienced appreciation is based on individual performance and achievements but also represents the feeling of being evaluated as a human being with one's personality, habits, and self. We believe that appreciation should not be understood as one resource among others that could be conserved or used to buffer specific demands, but as an influential factor that reaches deeper by strengthening or threatening the individual's self-esteem and, therefore, fulfilling or endangering a basic human need. This is why we decided to build EA-SI based on the SOS theory. For a more diversified discussion of other theories in contrast to the SOS theory, please see Semmer et al. (2019).

#### 2.3.2 The SOS theory—EA-SI and self-esteem

A core assumption of the SOS theory is that protecting and maintaining a positive self-esteem represents a basic human need (Semmer et al., 2007, 2019). *Self-esteem* includes social and personal components (Lazarus, 2006; Semmer et al., 2019). Personal self-esteem focuses on self-evaluation and acknowledgment of one's worth. Social self-esteem involves actions, feedback, and evaluation provided by the social environment (Semmer et al., 2019) relating to employees' work-associated identities (Pfister et al., 2020). Both types of self-esteem are interrelated (Semmer et al., 2019).

Since it is a basic need to protect the self-esteem, there are specific threats or boosts to it (Semmer et al., 2007). Boosts are actions or environmental sensations—felt at work—that reflect positive evaluations of performance or personality and, therefore, strengthen one's self-esteem. Threats to the self are negative or devaluating messages received directly or indirectly from one's (work) environment (Semmer et al., 2019). In line with De Cremer and Tyler (2005)—who showed that respectful intragroup interactions can strengthen but also threaten work-related identity depending on their expression—we assume that EA-SI can function as a boost but also as a threat to employees' self-esteem if absent.

The work context is one of the most important and identity-shaping environments within individuals' lives (Ashforth and Schinoff, 2016). Since the personal and social self-esteem are intertwined, we expect experienced appreciation to be related to employees' global self-esteem, including social and personal aspects in a single construct (von Collani and Herzberg, 2003). The *global self-esteem* is based on the overall self-evaluation of one's personal worth, traits, and personality (Brown, 2010; Schütz and Röhner, 2025). Higher global self-esteem favors a more self-loving attitude toward oneself, buffering the negative impact of being confronted with failure (Brown, 2010). The global self-esteem is more driven by personal evaluation than objective parameters. Nonetheless, the subjective evaluation of one's self is strongly influenced by the social environment and the assessment through others (Leary and Baumeister, 2000). Consequently, we assume that experienced appreciation at work does not influence one aspect of employees' self-esteem but their global self-esteem.

**H1a:** The more employees feel appreciated by their colleagues, the higher their self-esteem.
**H1b:** The more employees feel appreciated by their direct supervisors, the higher their self-esteem.

#### 2.3.3 The SOS theory—EA-SI and stress

Threats to the self-result in a stress reaction (Semmer et al., 2019). The SOS theory differentiates two forms of stress, stress through insufficiency (SIN) and stress as disrespect (SAD). SIN occurs when individuals perceive themselves, their abilities, and their achievements as falling short of self-defined goals. SAD results from interactions with others that lead to feelings of inadequacy or devaluation (Semmer et al., 2019).

We believe that EA-SI at work influences both forms of stress. Appreciation at work influences whether employees feel treated respectfully and—understood as feedback from others—determines whether they feel sufficient to master demands. The sensation of feeling insufficient should be influenced by the evaluation from and actions of others, and vice versa. We believe that (non-)appreciative experiences at work influence employees' global self-esteem and general stress perception. The *general stress perception* combines the dimensions of worries, tension, demands, and joy in one construct (Fliege et al., 2001). We believe that employees who have to defend against threats to their self-esteem—due to low levels of EA-SI at work—experience less joy and more worries, demands, and tension. To replicate the fundamental assumptions of the SOS theory, we also expect lower self-esteem to be related to an increased perception of stress (Pfister et al., 2020; Semmer et al., 2019; Stocker et al., 2014).

**H2a:** The less employees feel appreciated by their colleagues, the higher their stress perception.
**H2b:** The less employees feel appreciated by their direct supervisors, the higher their stress perception.
**H3:** The lower employees' self-esteem, the higher their stress perception.

#### 2.3.4 EA-SI and employee satisfaction

Experienced stress can result from low levels of experienced appreciation (Semmer et al., 2007) and relate to negative outcomes such as impaired wellbeing, health, and satisfaction (Siegrist, 2016; Stocker et al., 2010). Stress is also linked to depressive symptoms (Sakata et al., 2008), sleep disturbance, and fatigue (Fahlén et al., 2006). Chronic stress exposure relates to higher blood pressure, higher levels of cortisol, and an elevated risk of coronary diseases (Marmot et al., 2002).

Al-Aali and Ahmed (2022) found a negative correlation between perceived stress (in terms of an imbalance between effort and reward) and employee work satisfaction. In line with this, low levels of appreciation at work—as a stress-inducing factor—also predicted lower work satisfaction (Pfister et al., 2020; Semmer et al., 2005). *Satisfaction with work* reflects the subjective evaluation of recent professional activities and the organizational environment compared to personal expectations and needs (Irawanto et al., 2021). Consequently, high satisfaction with work should occur when the desired state at work aligns with the actual environment and demands. Hence, we expect employees who feel appreciated to be more satisfied at work.

**H4a:** The more employees feel appreciated by their colleagues, the higher their job satisfaction.
**H4b:** The more employees feel appreciated by their direct supervisors, the higher their job satisfaction.

We do not understand employees' work lives and the appreciation they do or do not receive at work as unrelated to other aspects of their lives. On the contrary, we believe that sensations at work spill over into other aspects of one's life. Examples of spillover are work-family conflicts, describing specific events from the work sector that impact employees' lives outside their work (Cao et al., 2020; Wolfram and Gratton, 2014). This spillover can result in positive and negative outcomes. For example, negative events at work impair sleep quality (Liu et al., 2020), while positive events relate to greater life satisfaction (Stevanovic and Rupert, 2009). Moreover, (Yildirim et al. 2024) showed that the experience of a meaningful life mediated the relation between occupational stress and satisfaction at work. Hence, we believe that EA-SI does not solely influence employees' job satisfaction but should also—associated with higher self-esteem and reduced perception of stress—relate to their overall life satisfaction.

The individual's wellbeing includes cognitive and emotional components (Keyes et al., 2002). In this article, we focus on employees' *satisfaction with life*, defined as the cognitive evaluation of whether a person's life meets their personal standards, expectations, and preferences (Diener et al., 1985). In line with the expected spillover between different areas of employees' lives (Lourel et al., 2009), we assume work and life satisfaction to be interrelated (Rain et al., 1991).

**H5a:** The more employees feel appreciated by their colleagues, the higher their life satisfaction.
**H5b:** The more employees feel appreciated by their direct supervisors, the higher their life satisfaction.
**H6:** The more satisfied employees are with their work, the higher their overall life satisfaction.

#### 2.3.5 EA-SI and employee motivation

To further evaluate EA-SI's criterion validity, we examined whether experienced appreciation relates to employee engagement at work. *Work engagement* characterizes the feeling of energy, inspiration, and motivation regarding one's work (Schaufeli et al., 2006). Work engagement is linked to employees' health and organizational commitment (Christian and Slaughter, 2007), as well as to higher work satisfaction (Mazzetti et al., 2023) and decreased burnout (Maricuţoiu et al., 2017). Meta-analytic evidence showed that job resources—present at group- and leader-level—can increase work engagement (Lesener et al., 2019). In line with these findings, higher appreciation at work is also related to higher job-related motivation (DiPietro et al., 2014) and, in terms of adequate reward, benefits work engagement (Al-Aali and Ahmed, 2022). Thus, we derive the following hypotheses.

**H7a:** The more employees feel appreciated by colleagues, the higher their work engagement.
**H7b:** The more employees feel appreciated by direct supervisors, the higher their work engagement.

#### 2.3.6 EA-SI and employee wellbeing

**Figure 1:**
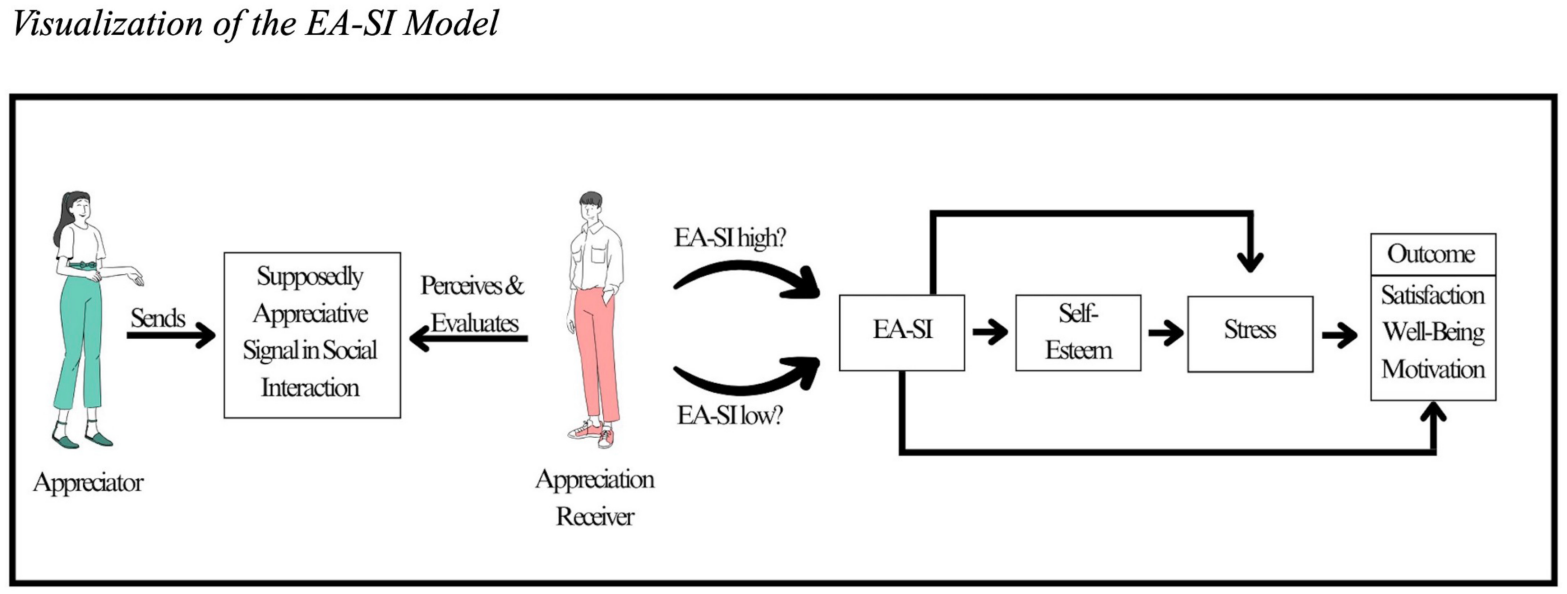
Visualization of the EA-SI model. Note. The model combines EA-SI with the SOS theory and basic considerations of communication.

Emotional exhaustion represents one of three dimensions of burnout (Korczak et al., 2010). The other two facets are loss of meaning (Wörfel et al., 2015) and a feeling of reduced performance (Korczak et al., 2010). Emotional exhaustion encompasses feelings of fatigue, the perception of insurmountable demands, and an impaired ability to emotionally connect to others (Korczak et al., 2010). It is related to stress (Jiménez-Ortiz et al., 2019) and represents an important measure of employee wellbeing (Crawford et al., 2010). Emotional Exhaustion is related to stress (Teo et al., 2021), dysregulation of the hypothalamic stress axis, chronic inflammation, and poor sleep quality (Melamed et al., 2006). In turn, resourceful social interactions at work are associated with less burnout (Maslach and Leiter, 2017). To test for criterion validity, we assume that EA-SI—related to employees' stress perception—should also be negatively related to emotional exhaustion. Figure 1 sums up the EA-SI model.

**H8a:** The more employees feel appreciated by colleagues, the lower their emotional exhaustion.
**H8b:** The more employees feel appreciated by direct supervisors, the lower their emotional exhaustion.

## 3 Study one

We designed the first study to revise the operationalization of EA-SI. Therefore, we theoretically derived a first draft of the EA-SI Work Scale. This draft was evaluated for its content validity and tested in a branch-heterogeneous sample against established psychometric standards in scale construction (Bortz and Döring, 2006; Moosbrugger and Kelava, 2008; Pospeschill, 2013). Based on all criteria, further elaborated in Section 3.1.6, we developed the revised EA-SI Work Scale and explored its reliability and dimensional structure.

In addition to scale construction, the first study serves as a pilot to validate the EA-SI Work Scale. Hence, the scale's criterion validity was evaluated in additional correlation analyses. Please note that we will use the term “EA-SI” to refer to the theoretical construct, while we will use the scale's full name when referring to the construct's operationalization.

### 3.1 Method—study one

#### 3.1.1 Transparency and openness

We described all measures and transparently reported data exclusion and manipulation. We used JASP 0.18.3 (JASP Team, 2024) and R (R version 4.4.1, R Core Team, 2024) within the RStudio environment (Version 2024.12.1) to analyze the data. The analyses in R were performed using the “tidyverse” (vo.2.0.0; Wickham et al., 2019), “lavaan” (vo.06-19; Rosseel, 2012), “psych” (vo.4.6.26; Revelle, 2024) and “lm.beta” (vo. 1.7-2; Behrendt, 2023) packages. The processed data—referring to the revised EA-SI Work Scale—are publicly available via osf.io using the link https://osf.io/m8ucv/?view_only=cb8b7bb959cb468082c25d305b9e0ddb. All instruments of relevance to this article are included. No code was uploaded. This study was not preregistered. There is a preprint available via osf.io following the link https://doi.org/10.31219/osf.io/rztc9_v1. Please note that the current article has been updated substantially during the review process, clarifying several erroneous information and reworking essential details in distinction to the first preprint. Moreover, as a continuation of the current article and its insights, Resch et al. (2025) developed a short scale to measure EA-SI at work. Although the short scale has been published prior to the current article, it is chronologically built on it.

#### 3.1.2 Design and acquisition

In Study One, we used a cross-sectional, field-study design. To obtain a heterogeneous sample, we recruited participants in Germany using a variety of methods, including mail, phone, on-site recruitment, and flyers in Spring 2022. The online questionnaire was generated with SoSci Survey (Leiner, 2024). Participants were informed that participating in the survey was voluntary, that they could end their participation at any point, and that their data collection and analysis were anonymous. The study was approved by the ethics committee of the Johannes Gutenberg University Mainz.

#### 3.1.3 Sample

We conducted a power analysis with a significance level of *p* = 0.05 and a power of 0.95, limiting the alpha and beta error to a maximum of 5%. With a moderate correlation coefficient of *r* = 0.30 (Cohen, 1988), the analysis implied a sample size of *N* = 138 (Hemmerich, 2018).

We collected *N* = 422 cases. To be considered for the survey, participants had to be between the age of 18 and 79, have daily contact with colleagues and direct supervisors, and work at least 8 h per week.

To ensure data quality, we excluded surveys that had not been fully completed (*n* = 173), participants who reported in the honesty item that they were unconcentrated or unconscientious (*n* = 5), and participants who answered < 80% of the EA-SI Work Scale (*n* = 13).

The final sample included *N* = 231 participants. Of these, 64.9% were female, 34.6% were male, and 0.4% were non-binary. Ages ranged from 19 to 65 [*mean* (*M*) = 37.9, *standard deviation* (*SD*) = 14.62]. One-fourth (25.5%) reported working in temporary employment, 57.2% had permanent employment, and 17.3% worked as civil servants. The sample included a wide range of occupations. If grouping the participants descriptively, the recruited professions spread across the branches “education & social work” (e.g., teacher, social worker), “communication & finance” (e.g., manager, banker), “IT & engineering” (e.g., software developer, construction manager), “service industry” (e.g., receptionist, waitress), “science” (e.g., researcher, auxiliary scientist), “civil service” (e.g., police officer, career counselor), and “healthcare” (e.g., nurse, medical assistant).

#### 3.1.4 The EA-SI work scale

To investigate EA-SI for different groups of appreciators, we developed one scale for colleagues and one for direct supervisors as appreciators. The first draft of the EA-SI Work Scale included *k* = 112 items. Fifty-six items focused on experienced appreciation from colleagues. The remaining items focused on direct supervisors as appreciators. The items were derived by combining different manifestations of supposedly appreciative messages/actions based on various understandings of appreciation integrated in the EA-SI model. This extensive item set was developed to ensure that the revised instrument included a sufficient number of items that could be evaluated as content valid and non-redundant but also survived the selection process. We chose that procedure to ensure that all relevant aspects of EA-SI were covered, even if specific items had to be removed due to quality issues. The scale ranged from 1 (*does not apply*) to 10 (*does apply*), including a fallback option to indicate that the item did not apply to the individual's work reality at all. A sample item reads, “My colleagues/supervisors show me that they know and appreciate my strengths.”

#### 3.1.5 Validation instruments

The German Rosenberg Self-Esteem Scale measured global self-esteem with *k* = 10 items (von Collani and Herzberg, 2003). The scale ranges from 1 (*not at all*) to 6 (*completely*). A sample item is “[o]verall, I am satisfied with myself.”

The perceived stress level of participants was measured using the German version of the Perceived Stress Questionnaire with *k* = 20 items (Fliege et al., 2001, 2009). The four-point rating scale ranges from 1 (*almost never*) to 4 (*most of the time*). One sample item is “[h]ow often do the following statements apply … You feel tense.”

Workplace satisfaction was measured with *k* = 3 German items, referring to Baillod and Semmer (1994). The items could be answered on one of two different seven-point rating scales, ranging from 1 (*exceedingly unsatisfied*) to 7 (*exceedingly satisfied*) and from 1 (*hardly ever*) to 7 (*very often*). An example is “[h]ow satisfied are you in general with your work?”

We used *k* = 5 items of the German version of the Satisfaction With Life Scale (Janke and Glöckner-Rist, 2012) to assess life satisfaction. The seven-point Likert scale ranges from 1 (*strongly disagree*) to 7 (*strongly agree*). A sample item is “[i]n most ways, my life is close to my ideal.”

Using the Utrecht Work Engagement Scale-9 (UWES-9), we addressed work engagement with *k* = 9 items (Schaufeli et al., 2006). The items ranged from 1 (*never*) to 7 (*every day*). A sample item is “I am enthusiastic about my work.”

Referring to Korczak et al. (2010), we used *k* = 9 items of the Maslach Burnout Inventory, translated into German, to measure emotional exhaustion. We offered participants a seven-point rating scale from 1 (*never*) to 7 (*every day*). A sample item is “After working, I feel exhausted.”

To ensure consistency regarding the time for different measures, we instructed participants to reflect on the past 3 months. To eliminate order effects, we randomized the item presentation within the instruments. Additionally, Study One surveyed effort-reward imbalance, social support, turnover intention, various measures of appreciation, and employee absenteeism. Those measures are not relevant to this article.

#### 3.1.6 Statistical analyses

In the first step, we asked naïve and expert judges to evaluate the items' content validity as well as their verbal and formal redundancy. Subsequently, we tested the item set with *k* = 56 for each group of appreciators against established quality standards in instrument construction (Moosbrugger and Kelava, 2008; Pospeschill, 2013) to revise the first draft of the EA-SI Work Scale. Following the described standards, all items with a skewness ≥2, an excess ≥7 (West et al., 1995), an item difficulty above 0.8 or below 0.2, and with a discriminatory power ≤ 0.3 (Bortz and Döring, 2006) were excluded. Additionally, we decided to exclude items that did not apply to the work of more than 20% of participants. This cutoff was defined based on a visual analysis of data distribution. On average, participants chose the fallback option considerably less frequently. In a third step, we synchronized the remaining items so that only the items that survived the selection process for both groups of appreciators were kept. Since we aimed to develop a versatile but also time-economic applicable scale, we decided that the time to answer the EA-SI Work Scale should not exceed 2 min for each group of appreciators. To determine the estimated response time, we used an established mathematical equation from market research that considered the word count, the number of decisions participants had to make, and the number of items (Puleston, 2012). The equation indicated a duration of *d* ~= 2 for a maximum of *k* = 15 items. The detailed formula can be found in Appendix A.

To investigate the dimensional structure of EA-SI, we conducted exploratory factor analyses (EFA) based on the revised EA-SI Work Scale. MacCallum et al. (1999) and Harlow (2014) recommended a sample size of between 200 and 400 participants for factor analyses. Based on this criterion, the sample size is adequate. We used the maximum likelihood method with orthogonal varimax rotation, scree plots, and parallel analysis (O'connor, 2000) to identify the number of extracted factors.

To test for criterion validity, we conducted Pearson product-moment correlations. To evaluate the strength of the correlations, we applied the cutoff values by Cohen (1988), assuming *r* ≥ 0.1 to indicate a small, *r* ≥ 0.3 a moderate, and *r* ≥ 0.5 a strong correlation. As a level of significance, we applied *p* < 0.05 (Fisher, 1956).

Since the data is based solely on self-reported measures, we tested for common method bias (CMB) in both studies. All analyses on CMB can be found in Appendix B. Referring to Fuller et al. (2016), in Study One, we tested for common method bias using Harman's single-factor test. Substantial common method bias should be present if a single factor would explain more than 50% of the variance (Fuller et al., 2016). Referring to the assumptions by Söhnchen (2009), we additionally computed a scree plot and parallel analysis to examine whether the exploratory factor analysis would imply a single-factor solution, also indicating substantially biased data.

### 3.2 Results

#### 3.2.1 Item selection

First, we excluded one item for both groups of appreciators that did not apply to the work of more than 20% of participants. We then eliminated *k* = 7 items for colleagues and *k* = 4 items for direct supervisors that had a skewness ≥2. In contrast, all items met the criteria of excess ≥7 (West et al., 1995). Third, we excluded *k* = 11 items for colleagues and *k* = 8 items for direct supervisors with an item difficulty above 0.8 or below 0.2. Finally, we eliminated *k* = 1 item for colleagues and *k* = 2 items for direct supervisors with a discriminatory power ≤ 0.3 (Bortz and Döring, 2006).

In the next step, we synchronized the item sets for both groups of appreciators. Therefore, we only kept the items that met the described psychometric criteria for both colleagues and direct supervisors. *k* = 3 items met the criteria for direct supervisors but not for colleagues, while *k* = 8 different items met the criteria for colleagues but not for direct supervisors. We eliminated these items so that both scales consisted of a comparable set of *k* = 34 items.

Considering all criteria, we decided to eliminate *k* = 19 additional items, with either low content validity, high verbal redundancy, or high formal redundancy. The *k* = 15 items of the revised EA-SI Work Scale can be found in Appendices C–F. In all future analyses, only the items of the revised instrument were considered.

#### 3.2.2 Factor structure of EA-SI—colleagues as appreciators

Since the total number of unanswered items was 4.4% and, therefore, well below the recommended 10% suitable for mean imputation (Watkins, 2018), we substituted missing values with the item's arithmetic mean prior to factor analyses. The Kaiser–Meyer–Olkin criterion (Kaiser, 1974; KMO) showed an overall sampling adequacy of *KMO* = 0.93, suggesting that the data were “marvelously” suitable for EFA (Watkins, 2018). Bartlett's test of sphericity (Bartlett, 1954) indicated a non-random data structure $\chi_{\left(105\right)}^{2}$ = 1626.96, *p* < 0.001. The scree plot and the parallel analysis indicated a one-dimensional solution for EA-SI (O'connor, 2000; Watkins, 2018). The model was significant *p* < 0.001. The Eigenvalue for the factor was 6.88. The model accounted for 42.4% of the variance.

#### 3.2.3 Factor structure of EA-SI—direct supervisors as appreciators

**Figure 2:**
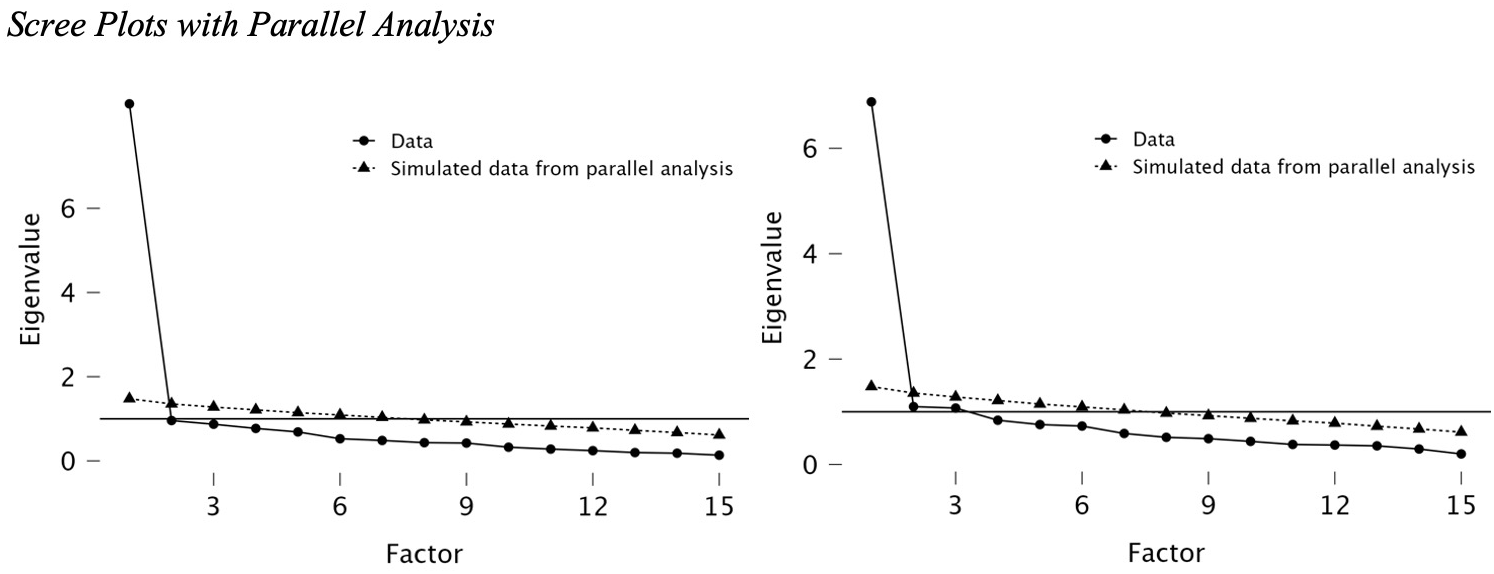
Scree plots with parallel analysis. Note. The left plot shows factor loadings and Analyses for colleagues, and the right for supervisors. Circular-shaped dots represent the original data, while triangular-shaped dots show the simulated values from the parallel analysis.

For direct supervisors, the *KMO* = 0.96, as well as Bartlett's test of sphericity $\chi_{\left(105\right)}^{2}$ = 2438.39, *p* < 0.001, identified the data as suitable for EFA (Watkins, 2018). The scree plot and parallel analysis implied a single-factor solution (O'connor, 2000; Watkins, 2018). The Eigenvalue was 8.48. The model was significant *p* < 0.001 and explained 53.8% of the item variance. Table 1 shows the factor loadings for all items separated by the groups of appreciators, while Figure 2 depicts the related scree plots and parallel analyses of the EFA.

**Table 1:** Exploratory factor analyses EA-SI work scale separated by groups of appreciators—study one.

**Item**	**Factor 1**	**Uniqueness**	**Item**	**Factor 1**	**Uniqueness**
EA-SI_1_coll	0.86	0.27	EA-SI_1_sup	0.91	0.18
EA-SI_2_coll	0.84	0.30	EA-SI_2_sup	0.88	0.22
EA-SI_3_coll	0.80	0.40	EA-SI_3_sup	0.88	0.23
EA-SI_4_coll	0.74	0.45	EA-SI_4_sup	0.85	0.27
EA-SI_5_coll	0.72	0.49	EA-SI_5_sup	0.85	0.28
EA-SI_6_coll	0.72	0.49	EA-SI_6_sup	0.84	0.29
EA-SI_7_coll	0.68	0.54	EA-SI_7_sup	0.79	0.38
EA-SI_8_coll	0.64	0.59	EA-SI_8_sup	0.78	0.40
EA-SI_9_coll	0.62	0.62	EA-SI_9_sup	0.72	0.48
EA-SI_10_coll	0.60	0.64	EA-SI_10_sup	0.67	0.56
EA-SI_11_coll	0.52	0.73	EA-SI_11_sup	0.64	0.59
EA-SI_12_coll	0.50	0.76	EA-SI_12_sup	0.63	0.61
EA-SI_13_coll	0.50	0.76	EA-SI_13_sup	0.48	0.77
EA-SI_14_coll	0.45	0.79	EA-SI_14_sup	0.43	0.82
EA-SI_15_coll	0.42	0.83	EA-SI_15_sup	0.38	0.86

The left part shows factor loadings for colleagues the right part for direct supervisors.

#### 3.2.4 Testing for common method bias

Harman's single-factor test indicated that a one-factor solution would explain 31.5% of the variance. The computed scree plot and parallel analysis implied a six-factor solution. Since these results imply no substantial evidence for common method bias, all analyses are computed without further controlling for CMB.

#### 3.2.5 Validation of the EA-SI work scale—criterion validity

As predicted, EA-SI was positively correlated with employees' global self-esteem (colleagues: *r* = 0.36, *p* < 0.001; direct supervisors: *r* = 0.35, *p* < 0.001) and negatively correlated with perceived stress (colleagues: *r* = −0.46, *p* < 0.001; direct supervisors: *r* = −0.53, *p* < 0.001). In line with the SOS theory, global self-esteem and perceived stress were negatively correlated with each other (*r* = −0.55, *p* < 0.001).

EA-SI was positively related to work satisfaction (colleagues: *r* = 0.47, *p* < 0.001; direct supervisors: *r* = 0.55, *p* < 0.001) and life satisfaction (colleagues: *r* = 0.30, *p* < 0.001; direct supervisors: *r* = 0.31, *p* < 0.001) as well as to work engagement (colleagues: *r* = 0.41, *p* < 0.001; direct supervisors: *r* = 0.49, *p* < 0.001). EA-SI was negatively related to emotional exhaustion (colleagues: *r* = −0.52, *p* < 0.001; direct supervisors: *r* = −0.56, *p* < 0.001). Employee work satisfaction was positively correlated with life satisfaction (*r* = 0.35, *p* < 0.001). Table 2 summarizes the mean, standard deviation, internal consistency, and correlations of all instruments.

**Table 2:** Pearson product-moment correlations—study one.

**Constructs**	**M**	**SD**	**α**	**1**	**2**	**3**	**4**	**5**	**6**	**7**	**8**
1. EA-SI—colleagues	7.59	1.51	0.91	–							
2. EA-SI—supervisors	7.26	2.01	0.94	0.64^**^	–						
3. Self-esteem	5.05	0.88	0.90	0.36^**^	0.35^*^	–					
4. Perceived stress	2.23	0.67	0.94	−0.46^**^	−0.53^**^	−0.55^**^	–				
5. Workplace satisfaction	4.76	1.55	0.76	0.47^**^	0.55^**^	0.29^**^	−0.55^**^	–			
6. Life satisfaction	5.26	1.16	0.88	0.30^**^	0.31^**^	0.56^**^	−0.59^**^	0.35^**^	–		
7. Work engagement	5.10	1.35	0.94	0.41^**^	0.49^**^	0.37^**^	−0.51^**^	0.67^**^	0.39^**^	–	
8. Emotional exhaustion	2.76	1.42	0.92	−0.52^**^	−0.56^**^	−0.43^**^	0.75^**^	−0.64^**^	−0.40^**^	−0.42^**^	–

Mean (M), standard deviation (SD), and Cronbach's alpha (α) are displayed. All correlations were tested one-sided with N = 231 participants. ^*^p < 0.05, ^**^p < 0.01.

## 4 Study Two

The second study included two time points of measurement. Study Two aimed to (1) replicate the unidimensional factor structure of EA-SI in a larger, independent sample, to (2) test the construct, (3) criterion, and (4) incremental validity of the EA-SI Work Scale, and to (5) investigate its test-retest reliability over a period of 4 weeks.

To investigate the construct validity of EA-SI, we added instruments to measure interpersonal justice, appreciation, workplace ostracism, and political beliefs. Social support served as a control variable when testing the criterion validity. Since we did theoretically derive appreciation and social support but have yet to define the other constructs, we will briefly explain them.

*Interpersonal justice* can be understood as the part of interactional justice, focusing on the extent to which employees feel treated respectfully and in an affectively sensitive way at work (Colquitt, 2001). Since one aspect of EA-SI addresses feelings of being heard and treated with respect, we expect EA-SI to be positively related to interpersonal justice. Together with alternative instruments to measure appreciation, interpersonal justice was implemented to test for EA-SI's convergent validity.

Furthermore, we examined *workplace ostracism*, defined as the sensation of being left behind or excluded at work (Ferris et al., 2008). Since EA-SI addresses the feeling of connecting and emotionally bonding with others, we expect a negative relation to experiencing exclusion at work. This is why we implemented workplace ostracism to test for discriminant validity.

Additionally, we asked participants about their *political opinions* regarding recent environmental decisions made by the German government. Referring to Ferris et al. (2008)—implying that this instrument is suitable as a measure of discriminant validity—we expect that the personal evaluation of current environmental politics should not be related to experienced appreciation at work.

### 4.1 Method—Study Two

#### 4.1.1 Transparency and openness

We described all measures and transparently reported data exclusion and manipulation. We again used the same versions and packages of the software JASP and R as in Study One. The processed data is publicly available via the link https://osf.io/m8ucv/?view_only=cb8b7bb959cb468082c25d305b9e0ddb. All surveyed instruments that are relevant to this article are reported. There is no uploaded code. This study was not preregistered. Please note that the sample and data described in the following section were also used by Resch et al. (2025) to initially derive the short scale based on the confirmatory factor analyses of the EA-SI Work Scale. The short scale was then validated in another, independent sample (Resch et al., 2025).

#### 4.1.2 Design and acquisition

The second study used a longitudinal, field-study design with two measurement points in the autumn of 2022. The two surveys were 4 weeks apart. To obtain a heterogeneous sample, we recruited participants in Germany using a variety of methods, including mail, phone, on-site recruitment, and flyers. We collected data using an online questionnaire generated with SoSci Survey (Leiner, 2024) available via internal servers of the Johannes Gutenberg University. Participants were informed that participating in the survey was voluntary, that they could end their participation at any point, and that their data collection and analysis were anonymous. The ethics committee of the Johannes Gutenberg University approved the study.

#### 4.1.3 Sample

For a regression analysis with two predictors and a moderate *R*^2^ = 0.13 (Cohen, 1988), the power analysis implied a sample size of *N* = 107 (Hemmerich, 2019). The applied significance level was *p* = 0.05, and the implemented power was 0.95.

At the first time point of measurement (T1), we collected *N* = 541 cases. We applied the same exclusion criteria as in the first study. Therefore, we excluded all incomplete datasets (*n* = 131), those who reported not answering concentrated or conscientious (*n* = 15), and (*n* = 4) surveys with < 80% of answered items within the EA-SI Work Scale. The final sample at T1 included *N* = 391 participants. Of those, *n* = 260 (66.5%) were female, *n* = 129 (33%) were male, and *n* = 2 (0.5%) identified as non-binary. The youngest participant was 18, the oldest was 71 years old (*M* = 34.04, *SD* = 13.67). More than one-third (34.5%) worked in temporary employment, 51% were permanently employed, 12.8% worked in civil service, and 1.7% worked as freelancers.

At the second point of measurement (T2), we collected *N* = 203 surveys. We excluded *n* = 43 participants who did not complete the questionnaire, *n* = 3 who did not answer the survey concentrated and/or honestly, *n* = 13 with more than 20% of missing values in the EA-SI Work Scale, and *n* = 11 data sets that were not matchable with T1. The final sample of T2 consisted of *N* = 134 participants. Study Two also included a multitude of different professions, spread across the same branches as named in Study One.

#### 4.1.4 Instruments

In Study Two, we again examined EA-SI, self-esteem, perceived stress, work and life satisfaction, work engagement, and emotional exhaustion. We used the same instructions and randomized item presentation as in Study One. To investigate the construct and incremental validity of EA-SI, we included additional instruments, which will be described in the following section.

We implemented three more instruments to measure appreciation at work. The Appreciation at Work Scale (AAWS; Jacobshagen et al., 2008) measures appreciation with *k* = 5 items for colleagues and *k* = 5 items for supervisors. The scale ranges from 1 (*does not apply at all*) to 7 (*does fully apply*). A sample item reads, “[m]y supervisor praises me when I do my tasks well.”

Moreover, we included two additional single-item measures, translated into German, to address appreciation. One item was “[a]ltogether, how satisfied are you with the appreciation you receive from your supervisor?” (Jacobshagen et al., 2008), using a scale ranging from 1 (*extremely unsatisfied*) to 7 (*extremely satisfied*). The second item was “[d]o your colleagues value your work?” (Bakker et al., 2007), applying a scale from 1 (*hardly ever*) to 4 (*very often*).

To measure interpersonal justice, we translated *k* = 4 items referring to Colquitt (2001) into German and adapted them to focus on colleagues and direct supervisors as sources of interpersonal justice. The scale ranges from 1 (*seldom*) to 5 (*often*). A sample item is “[d]id your colleagues treat you in a polite way?”

To measure workplace ostracism, we translated *k* = 3 of the items of the Workplace Ostracism Questionnaire by Ferris et al. (2008) into German. The seven-point rating scale ranged from 1 (*never*) to 7 (*always*). One item was “[o]thers ignored you at work.”

Participants' attitudes toward the current German environmental policy were measured with *k* = 4 translated items in analogy to (Ferris et al. 2008). Each question used the item stem “[h]ow do you rate the current environmental policy of your government regarding the following qualities …” Participants rated the policy using four differential scales ranging from 1 (*bad/foolish/harmful/useless*) to 5 (*good/wise/beneficial/useful*).

In analogy to the Social Support Scale (Frese, 1989), we included *k* = 5 adapted items focusing on colleagues and *k* = 5 items focusing on direct supervisors on a four-point Likert scale ranging from 1 (*not at all*) to 4 (*absolutely*). A sample item is “[h]ow much can you rely on your direct supervisors when things get difficult at work?”

In addition, we surveyed personality traits, turnover intention, effort-reward imbalance, sleep quality, employee absenteeism, and presenteeism. Those instruments are of no relevance to this article.

#### 4.1.5 Statistical analyses

First, we examined the factor structure of the EA-SI Work Scale with confirmatory factor analyses (CFA) to test its one-dimensional structure. Since the χ^2^-Test of model fit is sensitive to sample size and model complexity (Kline, 2023), we used the additional fit indices *Comparative Fit Index* (CFI), *Standardized Root Mean Square Residual* (SRMR), and *Root Mean Square Error of Approximation* (RMSEA) to determine the fit of the expected one-factor solution. A CFI > 0.95 (Hu and Bentler, 1999), SRMR < 0.08 (Byrne, 2013), and RMSEA < 0.08 (Awang, 2012) indicate a good model fit. We further implemented the CFI and SRMR since they are—in contrast to the RMSEA—robust to the number of degrees of freedom (Chen et al., 2008).

In addition to the χ^2^-Test, Kenny et al. (2015) recommended to consider the residual covariance matrices or misfit plots to evaluate the global model fit of the factor solution. These matrices depict the dyadic residual variance between the single items of the instrument. A poor fit of the model is indicated in case of high values (Kenny et al., 2015). Following these recommendations, we analyzed the misfit plots for both groups of appreciators. According to Rogers (2024) misfit plots with values (predominantly) below the cutoff of 0.10 indicate a “good” model fit.

To test for common method bias, we applied Harman's single-factor test (Fuller et al., 2016). As in Study One, an implied single-factor solution explaining more than 50% of the variance should indicate common method bias (Fuller et al., 2016; Söhnchen, 2009). Since Harman's single-factor test has been controversially discussed for several limitations (Podsakoff et al., 2003), we additionally applied the marker variable technique by Lindell and Whitney (2001). This method recommends the inclusion of a theoretically unrelated instrument that “can be used as a marker in that any observed relationships between it and any of the other variables can be assumed to be due to common method variance” (Podsakoff et al., 2003, p. 893). As a marker variable, we chose participants' environmental policy attitude, since we did not expect it to be related to our substantive variables while sharing the same Likert scale format, possibly making it susceptible to “comparable response processes and tendencies” (Simmering et al., 2015). This type of marker should represent an “ideal marker” (Richardson et al., 2009). Referring to Lindell and Whitney (2001), to test for CMB, we chose the smallest positive correlation coefficient between the criterion and the marker variable to compute partial correlations.

To test whether EA-SI fulfills the requirements of a construct-valid instrument, we conducted Pearson product-moment correlations with convergent and discriminant measures. High construct validity is given if EA-SI is positively correlated with convergent measures and negatively or not correlated with discriminant measures (Moosbrugger and Kelava, 2008).

The criterion validity was tested by correlatively replicating the assumptions of the SOS theory. Appendix G shows the internal consistency, mean, standard deviation, and correlations regarding all relevant measures. To evaluate the strength of the Pearson product-moment correlations, we applied the same cutoff values as in Study One (Cohen, 1988).

Moreover, we conducted hierarchical regression analyses—for both groups of appreciators and controlled for social support—to test for EA-SI's incremental criterion validity. Social support was included in the first model to examine the additional predictive value of EA-SI. The standardized regression coefficients were computed to evaluate the predictive value of EA-SI and social support regarding the described outcome measures. To determine the effect size of the overall model, we computed the adjusted *R*^2^. Referring to Cohen (1988) we interpret *R*^2^ ≥ 0.02 as a small, *R*^2^ ≥ 0.13 moderate, and *R*^2^ ≥ 0.26 strong effect size.

We then investigated the test-retest reliability for T1 and T2. Due to the 48% dropout rate between the two time points, we had to ensure that T1 and T2 were comparable and that there were no systematic differences between the two samples. Hence, we tested both time points for equivalence (Lakens, 2017) prior to reliability testing. Lower and upper bounds were defined as Δ_*L*_ = −0.5 and Δ_*U*_ = 0.5 (Lakens et al., 2018). As a level of significance, we applied *p* < 0.05 (Fisher, 1956).

### 4.2 Results

#### 4.2.1 Validation of EA-SI—factorial validity

We conducted two confirmatory factor analyses, one for colleagues and one for supervisors. While the χ^2^-Test indicated a poor fit for colleagues χ^2^(*N* = 391; *df* = 90) = 180.65, *p* < 0.001 and direct supervisors χ^2^(*N* = 391; *df* = 90) = 231.66, *p* < 0.001 the additional fit indices pointed toward excellent model fit for colleagues CFI = 0.97, RMSEA = 0.05, SRMR = 0.03 as well as for direct supervisors CFI = 0.97, RMSEA = 0.06, SRMR = 0.03. The additionally computed misfit plots supported the global model fit of the single factor solution. Table 3 presents all fit indices. The misfit plots are depicted in Appendix H.

**Table 3:** Results of the confirmatory factor analyses—study two.

**Measures of fit**	**Colleagues**	**Direct supervisors**
χ^2^	180.65	231.66
*df*	90	90
*p*-value	< 0.001	< 0.001
CFI	0.97	0.97
RMSEA	0.05	0.06
90% CI	[0.04; 0.06]	[0.05; 0.07]
SRMR	0.03	0.03

N = 391 participants. The 90% CI describes the confidence interval of RMSEA.

#### 4.2.2 Validation of EA-SI—testing for common method bias

Harman's single-factor test showed an explanatory power of 26.3% for the one-factor solution. Following the screeplot and parallel analysis, the extraction of 11 factors was suggested, indicating no substantial common method bias. In line with these findings, partialling out the smallest positive coefficient between the criterion and marker variable did not impact the significance of our results. Since none of the analyses implied substantial common method bias, all further analyses were conducted and interpreted without including the marker variable. As mentioned before, all tests on common method bias are depicted in Appendix B.

#### 4.2.3 Validation of EA-SI—construct validity

The EA-SI Work Scale was positively correlated with the AAWS (colleagues *r* = 0.70, *p* < 0.001; direct supervisors *r* = 0.84, *p* < 0.001) and with the single items (colleagues *r* = 0.66, *p* < 0.001; direct supervisors *r* = 0.79, *p* < 0.001). EA-SI was positively correlated with interactional justice (colleagues *r* = 0.37, *p* < 0.001; direct supervisors *r* = 0.50, *p* < 0.001) and negatively related to workplace ostracism (colleagues *r* = −0.27, *p* < 0.001; direct supervisors *r* = −0.22, *p* < 0.001). EA-SI did not relate significantly to employees' political opinions (colleagues *r* = −0.06, *p* = 0.223; direct supervisors *r* = −0.05, *p* = 0.305).

#### 4.2.4 Validation of EA-SI—criterion validity

First, we investigated the premises of the SOS theory with Pearson product-moment correlations. As theoretically assumed, EA-SI was related to employees' stress perception (colleagues *r* = −0.36, *p* < 0.001; direct supervisors *r* = −0.35, *p* < 0.001) as well as to self-esteem (colleagues *r* = 0.26, *p* < 0.001; direct supervisors *r* = 0.24, *p* < 0.001). Lower self-esteem related to higher perceived stress (*r* = −0.56, *p* < 0.001).

#### 4.2.5 Incremental criterion validity—colleagues as appreciators

EA-SI predicted higher work-related satisfaction (β = 0.47, *t* = 7.93, *p* < 0.001) above social support (β = 0.11, *t* = 1.95, *p* = 0.05), *R*^2^ = 0.28, *F*_(2, 388)_ = 78.39, *p* < 0.001 as well as higher employee life satisfaction (β = 0.20, *t* = 3.03, *p* = 0.002) above social support (β = 0.15, *t* = 2.23, *p* = 0.026), *R*^2^ = 0.09, *F*_(2, 388)_ = 21.26, *p* < 0.001. The more employees felt appreciated, the more engaged they were with their work (β = 0.47, *t* = 7.90, *p* < 0.001), social support was not a significant predictor after including EA-SI (β = 0.01, *t* = 0.23, *p* = 0.81), *R*^2^ = 0.23, *F*_(2, 388)_ = 58.33, *p* < 0.001. EA-SI did not predict lower emotional exhaustion (β = −0.11, *t* = −1.66, *p* = 0.09) above social support (β = −0.29, *t* = −4.67, *p* < 0.001), *R*^2^ = 0.14, *F*_(2, 388)_ = 31.66.

#### 4.2.6 Incremental criterion validity—direct supervisors as appreciators

Higher levels of experienced appreciation related to greater work satisfaction (β = 0.51, *t* = 7.97, *p* < 0.001) above social support (β = 0.10, *t* = 1.46, *p* = 0.13), *R*^2^ = 0.35, *F*_(2, 388)_ = 103.7, *p* < 0.001. EA-SI did not predict employees' overall life satisfaction (β = 0.11, *t* = 1.47, *p* = 0.14) beyond social support (β = 0.19, *t* = 2.44, *p* = 0.02), *R*^2^ = 0.08, *F*_(2, 388)_ = 16.79, *p* < 0.001. EA-SI predicted work engagement (β = 0.478, *t* = 6.85, *p* < 0.001) above social support (β = 0.01, *t* = 0.21, *p* = 0.83), *R*^2^ = 0.23, *F*_(2, 388)_ = 60.45, *p* < 0.001. The more employees felt appreciated by their direct supervisors, the lower their emotional exhaustion (β = −0.21, *t* = −2.8, *p* 0.005) when controlled for by social support (β = −0.18, *t* = −2.47, *p* = 0.014), *R*^2^ = 0.14, *F*_(2, 388)_ = 30.41, *p* < 0.001.

#### 4.2.7 Test-retest reliability of EA-SI

For colleagues, the tests against the lower bound Δ_*L*_, *t*_(131)_ = 5.88, *p* < 0.001, and against the upper bound Δ_*U*_, *t*_(131)_ = 5.61, *p* < 0.001 were significant. For direct supervisors, the tests against lower Δ_*L*_, *t*_(131)_ = 5.01, *p* < 0.001, and upper bound Δ_*U*_, *t*_(131)_ = −6.48, *p* < 0.001 were also significant. The overall *t*-test showed no significant difference between T1 and T2 for colleagues *t*_(131)_ = 0.131, *p* = 0.896 and direct supervisors *t*_(131)_ = −0.731, *p* = 0.466. Hence, we investigated the test-retest reliability of EA-SI over 4 weeks. The test-retest reliability was *r* = 0.79, *p* < 0.001 for colleagues, and *r* = 0.84, *p* < 0.001 for direct supervisors.

## 5 General discussion

Across two studies, we defined, measured, and validated the construct Experienced Appreciation in Social Interactions and the instrument to operationalize it. EA-SI is based on grand, middle-range, and applied theory, integrating various understandings of appreciation in a single construct. It does not solely focus on one specific expression of appreciation but includes conditional (e.g., recognizing one's professional performance and achievements) and unconditional (e.g., valuing the living being behind work and performance) aspects. The assumed mechanism of action is based on the Stress as Offense to Self-theory (Semmer et al., 2019) while considering fundamental premises of interpersonal relation and communication (Schulz von Thun et al., 2014; Watzlawick et al., 2016). This article aimed to theoretically and statistically clarify experienced appreciation at work and to contribute to reducing incongruency and incomparability in researching appreciation. The EA-SI Work Scale showed high psychometric quality, reliability, and validity across two independent, branch-heterogenous samples. Subsequently, we will summarize and discuss our findings from both samples and derive implications for researchers and practitioners.

### 5.1 Discussion of the findings—research questions and hypotheses

#### 5.1.1 Reliability

For colleagues and direct supervisors, the internal consistency of the EA-SI Work Scale was α > 0.9 and, therefore, excellent (Blanz, 2021). We examined the instrument's reliability over 4 weeks. With *r* ≥ 0.8 the test-retest reliability for both groups of appreciators was good (Moosbrugger and Kelava, 2020).

#### 5.1.2 Factorial validity

The explorative factor analyses in Study One pointed toward the single-factor solution as the most suitable model to describe EA-SI's dimensional structure. We then tested this assumed single-factor solution in a second study using confirmatory factor analyses. Referring to the limitations of the χ^2^-Test—tending to erroneously reject fitting models with increasing sample size (Kline, 2023)—we evaluated the confirmatory analyses based on the combination of the χ^2^-Test and the recommended additional fit indices CFI, SRMR, and RMSEA as recommended by Alavi et al. (2020). Additionally, we followed the recommendations by Kenny et al. (2015), analyzing the misfit plots for both groups of appreciators. While the χ^2^-Test was significant, all additional fit indices and both misfit plots indicated a good to excellent model fit of the one-dimensional solution.

However, we agree with Xia and Yang (2019) who stated that a good model fit based on additional fit indices would mainly show that a specific scale optimization did work. Above the results of factor analysis, it should be essential to substantively reflect on whether the model could be further optimized or does appear applicable in the way indicated by the statistical analysis (Xia and Yang, 2019). Based on the theoretical deduction of EA-SI and previous findings supporting the idea of appreciation as an autonomous, unidimensional construct (Semmer et al., 2019), we conclude that it is substantively reasonable to assume the distinct manifestations of experienced appreciation to be ordered around one central factor. Combining all information, we conclude that the results imply that EA-SI should be treated as a single-dimensional construct. Coherently, the item with the most substantial factor loading for both groups of appreciators was “[m]y colleagues/supervisors show me that I am worth a lot.”

#### 5.1.3 Construct validity

EA-SI was positively correlated with measures of appreciation and interpersonal justice, indicating convergent validity. To address discriminant validity, we examined the correlations between EA-SI workplace ostracism and environmental policy attitude. As expected, EA-SI was negatively related to workplace ostracism and did not substantially relate to participants' political opinions. Hence, the results strengthen the construct validity of the EA-SI Work Scale.

#### 5.1.4 Criterion validity

EA-SI was significantly correlated with self-esteem and reduced stress perception. This was true for colleagues and direct supervisors within both samples. Self-esteem and employee stress perception were negatively correlated in both samples. The correlation coefficients were predominantly moderate to high (Cohen, 1988). These findings are in line with hypotheses 1–3 and the expected relations based on the SOS theory (Semmer et al., 2019).

The more appreciated employees felt, the higher their work and life satisfaction and work-related motivation were. Feeling more appreciated was related to less emotional exhaustion. Work and life satisfaction were positively related to each other. All correlations were significant and predominantly moderate to high in both samples and for both groups of appreciators. Hence, hypotheses 4–8 can be accepted.

#### 5.1.5 Incremental validity of EA-SI

Additionally, we investigated the EA-SI Work Scale's predictive value above social support to determine the incremental validity of the instrument. As expected, feeling appreciated by colleagues explained work satisfaction (with a strong effect size), work engagement (with a moderate effect size), and life satisfaction (with a small effect size) above social support. In line with our assumptions, feeling appreciated by direct supervisors explained work satisfaction (with a strong effect size), work engagement, and emotional exhaustion (with a moderate effect size), above social support.

Nonetheless, contrary to our expectations, EA-SI from direct supervisors was no longer a significant predictor of life satisfaction after controlling for social support. In addition, EA-SI from colleagues did not predict emotional exhaustion above social support.

A statistical explanation for the unexpected findings could be the strong correlation between social support and EA-SI, underestimating the effect of the separate predictors on the criterion and benefiting the erroneous rejection of a significant result due to multicollinearity (Cohen et al., 2003). Furthermore, the remarkably limited variance within the construct social support could also have biased the results of the regression analyses (Darlington, 1978).

When focusing on life satisfaction, it should be mentioned that social support and experienced appreciation were only marginally related to the criterion. Theoretically, these small correlates could be explained since life satisfaction should be fueled by numerous aspects and circumstances exceeding the two constructs we examined (Diener et al., 1985). This limited influence of both predictors could be a substantive explanation for the unexpected findings.

When focusing on emotional exhaustion, the results could indicate that there are substantial differences between the influence of appreciation separated by various sources. For example, feeling supported by one's colleagues seems to be suitable to buffer emotional exhaustion, while feeling appreciated by them does not add an incremental benefit. In contrast, feeling seen and valued by one's direct supervisors seems to incrementally benefit the reduction of emotional exhaustion above feeling supported by them. This substantive interpretation of the unexpected findings—ascribing EA-SI by direct supervisors an elevated role in predicting emotional exhaustion—has yet to be tested.

Referring to the hierarchical analyses, we conclude that the results strengthen the assumption that EA-SI goes beyond social support as a predictor for employee satisfaction, wellbeing, and motivation. However, despite our attempts to explain the deviating findings, future research should strive to further delineate EA-SI from other constructs.

#### 5.1.6 Research questions

This section will summarize the answers to the research questions that guided our work. Based on the introduction and the factor analyses, we can answer the first two research questions: EA-SI provides a congruent definition of appreciation, integrating multiple understandings in one construct while using the SOS theory as a theoretical foundation **(Q1)**. Although EA-SI can be shown in multiple ways, the statistical analyses indicated a one-dimensional structure for colleagues and direct supervisors **(Q2)**. The EA-SI Work Scale is a reliable and valid operationalization of this new construct, substantially relating to employee satisfaction, motivation, and wellbeing **(Q3)**. These relations were true for the work context but also for employees' global self-esteem, general stress perception, and satisfaction with life. Hence, the findings support the assumption of spillover dynamics between different areas of life. Work experiences—or more explicitly, appreciative interactions at work—seem to affect employees' lives outside work and vice versa **(Q4)**. These findings were confirmed in both branch-heterogeneous samples and were not limited to specific professions **(Q5)**. Finally, EA-SI provided incremental predictive value beyond social support, strengthening the understanding of experienced appreciation as an autonomous construct rather than a sub-facet of social support **(Q6)**.

### 5.2 Limitations and theoretical implications

Although we developed an elaborate and integrative theoretical EA-SI model, more than the cross-sectional design is needed to investigate causal effects. Future research should focus on longitudinal designs to better understand and further validate the assumed mechanisms of action of EA-SI.

In spite of using well-established instruments, randomly presenting the item, and implementing various scales with different ranges to counteract common method bias, our data is entirely based on self-reports. Therefore, we conducted Harman's single-factor test (Fuller et al., 2016), ruling out a single-factor solution for the measured constructs in both studies (Söhnchen, 2009) and applied the marker variable technique by Lindell and Whitney (2001) to control for common method bias. None of these tests implied substantially biased results due to common method variance. Nonetheless, future research should further investigate EA-SI and its relevance for employees' lives within designs based on a variety of methods (Podsakoff et al., 2003) to thoroughly rule out common method bias. For example, biological markers (e.g., stress markers), observational data (e.g., number of tasks well-performed), peer reports, or other data not based on self-reports (e.g., salary, number of promotions, key performance indicators) could be used.

While the branch-heterogeneity in both samples represents a strength of this article, improving the ecological validity of the results, a disproportionate distribution of gender (e.g., non-binary participants) and employment (e.g., freelancers) was included. Future research should strive to replicate the findings of this article in samples with various gender and employment distributions to further strengthen the generalizability of our conclusions. Since both samples were too small for subgroup analyses, future research should also question whether the effects of EA-SI vary in specific subgroups on the personal and organizational level.

Overall, the EFA and CFA, as well as the additional fit indices and misfit plots, substantially supported the unidimensional factor structure of EA-SI. Nonetheless, the results were partially ambiguous. Hence, the assumed single-factor solution has to be replicated and further investigated in independent samples.

As pointed out in previous studies, our results also point toward differences between the appreciation received by supervisors and the appreciation received by colleagues. While Bregenzer et al. (2022) showed that general appreciation at work should be more beneficial than appreciation from supervisors, our results are ambiguous. For example, appreciation from direct supervisors was stronger related to outcome measures than appreciation from colleagues. Contrary to these findings, EA-SI from direct supervisors did not predict employees' life satisfaction above social support, while EA-SI from colleagues did. The question arises whether there is a significant difference between the relevance of collegial appreciation and appreciation from supervisors regarding employees' lives. Future research should attempt to unravel the distinct groups of appreciators and their relevance for appreciation receivers. Moreover, EA-SI should be transferred to and examined in different groups of appreciators and types of relationships (e.g., family interactions, student-teacher interactions, or romantic relationships).

Since the message that is being sent by the appreciator has to be interpreted by the appreciation receiver (Schulz von Thun et al., 2014), the decoding process could be influenced by specific attributes of the appreciation receiver, such as their personality, attitude toward others, or personal beliefs. In line with this, previous findings showed that the individual stress reactivity (Bibbey et al., 2013), as well as the stress experience and coping strategies (Roloff et al., 2022), also varied substantially with specific personality traits. In addition to variables on the intrapersonal level, the relations between EA-SI and relevant outcome measures could also be influenced by variables on the interpersonal (e.g., leadership style) and/or organizational level (e.g., work surroundings, job complexity). Therefore, future research should control for potential mediators and/or moderators on these levels.

In addition, it should be noted that both studies are limited to German samples. In line with Triandis (2001)—indicating differences in psychological research between individuals with a collectivistic or individualistic worldview—future research should strive to replicate and validate EA-SI, controlling for culture-related differences.

Despite its benefits, measuring appreciation as a single-factor construct using all *k* = 15 items of the EA-SI Work Scale can be rather time-consuming. Therefore, based on the findings and limitations of the current article, Resch et al. (2025) developed a short scale to measure EA-SI, replicating and extending the knowledge about the construct in relation to employee satisfaction, engagement, and wellbeing. Hence, depending on the objective, researchers should weigh up the benefits and limitations of the long scale in contrast to the short scale and other alternatives when deciding which instrument to use.

### 5.3 Practical implications

We developed the EA-SI Work Scale for scientific purposes and practical application. The branch-heterogeneity of both samples, suggesting ecological validity (e.g., Kihlstrom, 2021), indicates practical relevance of experienced appreciation for jobholders across versatile professions. In addition, the predominantly moderate to large effect sizes further point toward the relevance of EA-SI for practical use.

The EA-SI Work Scale is characterized by psychometric quality and can easily be evaluated and interpreted by aggregating the answers in one arithmetic mean. The scale integrates conditional and unconditional expressions of appreciation in a theoretically integrative and statistically validated model. The definition of EA-SI—explicitly naming various expressions of appreciative behavior and messages—was designed to be comprehensible and easily applicable to foster organizational development.

Applying the understanding of experienced appreciation in social interactions as a reciprocal process of signals sent and decoded, organizations are encouraged to focus on both appreciators and appreciation receivers when fostering appreciative interactions at work. Hence, on the personal level, appreciators could be instructed on how to send appreciative messages with minimal risk of misunderstanding, while appreciation receivers could be enabled to perceive potential expressions of appreciation mindfully and to reflect on and communicate how they prefer to be appreciated.

On the organizational level, employers could (descriptively) derive specific implications from the results of the EA-SI Work Scale to increase appreciative interactions in their facilities. For example, organizations could implement training programs on professional communication and (active) listening, should the results imply that employees lack the feeling of being authentically listened to. Moreover, companies could offer team-building events and shared experiences, should the results imply a lack of reciprocal interest and/or opportunities to socially bond with each other. These are just two of the versatile examples of interventions that organizations could derive from the results of the EA-SI Work Scale.

Nonetheless, organizations should devise such interventions cautiously since they have not been experimentally evaluated, and the investigation of the scale's sensitivity to change is pending. Moreover, the possibility should be kept in mind that the targeted manipulation of one single manifestation of EA-SI may not be sufficient to substantially promote the entire construct. On the contrary, it could be possible that an appreciative culture on an overarching level could only be attained if all manifestations of EA-SI would exceed a specific threshold. However, the idea that a specific threshold must be overcome to accomplish a transition from one state to another is abstracted from research on mental states (e.g., Rouder and Morey, 2009) and has yet to be tested for EA-SI.

Considering the strong correlation between EA-SI and the Appreciation at Work Scale as well as the two single items, we recommend that organizations choose the instrument to measure appreciation based on their goals. Organizations interested in measuring appreciation in a more time-efficient way could consider shorter scales like the ones described in this article. Organizations aiming to measure experienced appreciation in a more diversified way—integrating various expressions of conditional and unconditional appreciation—could consider the EA-SI Work Scale.

Conclusively, we encourage professionals across different branches to spread and apply a coherent and comparable definition of appreciation at work. We hope that the construct “Experienced Appreciation in Social Interactions” and the EA-SI Work Scale contribute to countering the incongruency in defining and measuring appreciation and fostering a more appreciative culture at work.

## 6 Conclusion

Experienced Appreciation in Social Interactions is reliably and validly measurable. It is distinguishable from other constructs and explains outcomes above social support. EA-SI is relevant for employees' wellbeing, stress perception, self-esteem, satisfaction, and motivation. Our findings suggest that these relations are true across a variety of different occupations and areas of employees' lives. Therefore, the EA-SI Work Scale can be used by both researchers and practitioners to investigate experienced appreciation at work. Nonetheless, our findings are based on a newly developed construct and cross-sectional data. Future research is needed to better understand EA-SI and its mechanisms of action.

## Data Availability

The datasets presented in this study can be found in online repositories. The names of the repository/repositories and accession number(s) can be found in the article/Supplementary material.
