# Supplementary material for: Appreciation should be EA-SI—demystifying the definition and operationalization of experienced appreciation at work by developing a new construct
Source: Front Psychol. 2025 Sep 1;16:1445533. doi: 10.3389/fpsyg.2025.1445533 (PMC12434475; doi:10.3389/fpsyg.2025.1445533)
Supplement: Supplementary file 1 [file Table_1.pdf]

9 Appendices

9.1 Appendix A – Determination of the Estimated Response Time and Number of Items

$$\text{Duration} = (h(\text{words})/5 + h(\text{questions})*5 + (h(\text{decisions})-h(\text{questions}))*2 + h(\text{open questions})*15)/60$$

Note. The (estimated) number (*h)* of words, questions, and decisions must be implemented in the equation. Finally, the results of the equation have to be multiplied with the factor 1.05 if the survey is conducted in German language. This mathematical equation is based on the considerations by Puleston (2012).

9.2 Appendix B – Checking for Common Method Bias

Figure B1

Scree Plot with Parallel Analysis to Test for Common Method Bias – Study One

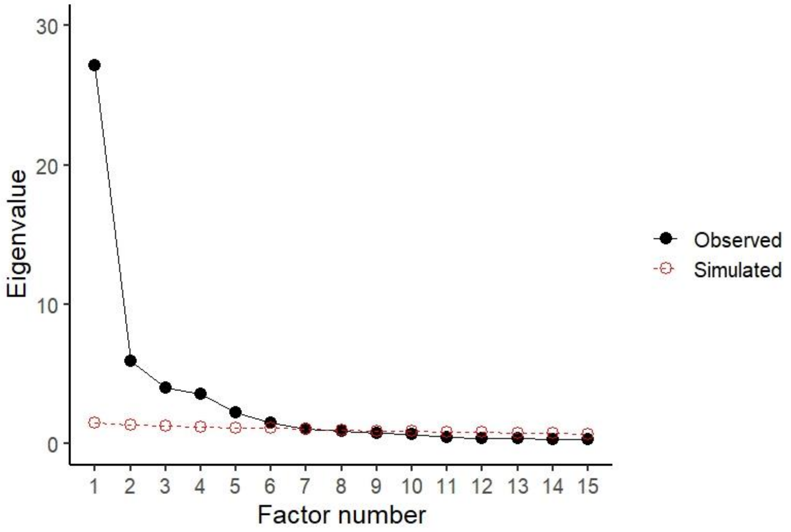

Figure B2

Scree Plot with Parallel Analysis to Test for Common Method Bias – Study Two

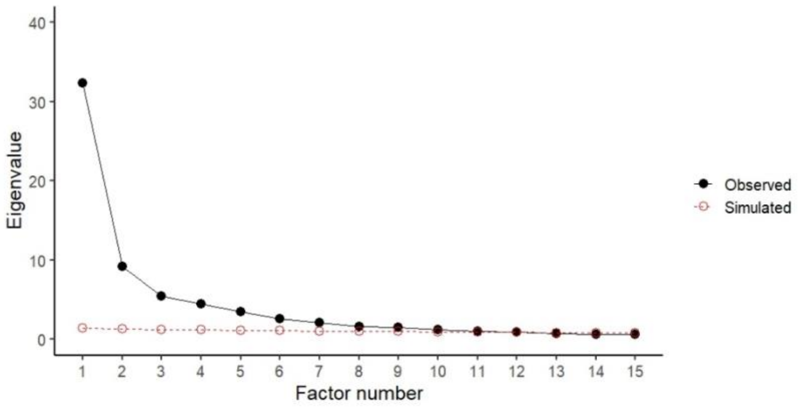

Table B1

Partial Pearson Product-Moment Correlations to Control for Common Method Bias – Study Two

	M	SD	α	1	2	3	4	5	6	7	8	9	10	11	12	13	14	15	16	17	18
1. EA-SI - Colleagues	7.02	1.52	.90	—																	
2. EA-SI - Supervisors	6.56	1.96	.93	.65**	—																
3. Self-Esteem	4.83	0.81	.89	.22**	.20**	—															
4. Perceived Stress	2.25	0.56	.93	-.43**	-.42**	-.64**	—														
5. AAWS - Colleagues	5.35	1.09	.85	.69**	.49**	.25**	-.34**	—													
6. AAWS - Supervisors	5.02	1.27	.88	.53**	.83**	.21**	-.46**	.52**	—												
7. Appreciation single - C	3.97	0.90	—	.65**	.47**	.19**	-.41**	.57**	.45**	—											
8. Appreciation single - S	5.19	1.63	—	.48**	.78**	.18**	-.48**	.40**	.78**	.49**	—										
9. Interactional Justice - C	4.47	0.68	.70	.33**	.24**	.22**	-.35**	.36**	.30**	.42**	.30**	—									
10. Interactional Justice - S	4.40	0.80	.79	.29**	.48**	.23**	-.40**	.24**	.52**	.34**	.58**	.64**	—								
11. Workplace Ostracism	1.49	0.64	.75	-.33**	-.29**	-.17**	.15**	-.38**	-.35**	-.40**	-.28**	-.50**	-.41**	—							
12. Marker Variable	3.53	0.80	.87	-.11	-.10	-.09	.09**	-.04	-.11	-.10	-.09	-.09	-.09	-.06	—						
13. Emotional Exhaustion	2.81	1.26	.90	-.37**	-.42**	-.48**	.65**	-.33**	-.45**	-.43**	-.48**	-.34**	-.42**	.32**	.00	—					
14. Work Engagement	4.69	1.44	.95	.45**	.46**	.23**	-.44**	.41**	.46**	.38**	.40**	.16**	.19**	-.08	-.10	-.38**	—				
15. Work Satisfaction	4.45	1.58	.84	.51**	.57**	.26**	-.52**	.43**	.53**	.49**	.56**	.28**	.34**	-.19**	-.11	-.54**	.71**	—			
16. Life Satisfaction	5.11	1.07	.87	.26**	.22**	.54**	-.59**	.24**	.25**	.23**	.20**	.17**	.18**	-.21**	-.12	-.45**	.32**	.33**	—		
17. Social Support - C	3.17	0.53	.82	.65**	.42**	.27**	-.41**	.62**	.42**	.56**	.38**	.36**	.28**	-.45**	.-.09	-.44**	.29**	.38**	.28**	—	
18. Social Support - S	2.99	0.69	.89	.41**	.76**	.23**	-.48**	.39**	.78**	.34**	.73**	.27**	.50**	-.31**	-.08	-.41**	.35**	.43**	.27**	.51**	—

Note. Mean (*M*), standard deviation (*SD*), and Cronbach's alpha (α) are displayed. All correlations were tested one-sided with a total $N = 391$. * $p<.05$, ** $p<.01$. All analyses are based on T1. Colleagues and supervisors are abbreviated with C and S. The marker variable refers to the variable partialized out of any observed relationship.

9.3 Appendix C – The EA-SI Work Scale (Colleagues)

Die folgenden Fragen beziehen sich auf die Wertschätzung, die Sie innerhalb Ihres Arbeitsalltages durch andere erfahren. Wichtig ist hierbei, dass es darum geht, wie sehr Sie sich persönlich wertgeschätzt fühlen. Es gibt also keine richtigen oder falschen Antworten. Bitte antworten Sie ehrlich und gewissenhaft. Uns interessieren an dieser Stelle Ihre persönlichen Empfindungen und Einschätzungen. Ihnen werden nachfolgend Aussagen präsentiert, die sich auf Ihre Kolleg:innen beziehen.

Bitte geben Sie an, inwieweit diese Aussagen auf Sie persönlich zutreffen. Erinnern Sie sich hierbei ganz gezielt an die letzten drei Monate. Bitte kreuzen Sie die Option „Kann ich nicht beantworten." nur dann an, wenn diese ganz und gar nicht für Ihre berufliche Tätigkeit zutreffend oder zu beantworten ist.

1. Meine Kolleg:innen nehmen meine Einwände ernst.
2. Meine Kolleg:innen zeigen mir, dass sie meine Stärken kennen und schätzen.
3. Häufig vertrauen mir meine Kolleg:innen Aufgaben an, die für das gesamte Team wichtig sind.
4. Meine Kolleg:innen betonen immer wieder, dass sie sich auf mich verlassen können.
5. Ich spüre deutlich, dass meine Kolleg:innen meine Arbeit zu schätzen wissen.
6. Ich fühle mich mit all meinen Erfolgen von meinen Kolleg:innen gesehen.
7. Ich erhalte angemessene ***materielle*** Geschenke (Pralinen, Blumenstrauß, Nahrungsmittel, kleiner Glücksbringer o.ä.) von meinen Kolleg:innen.
8. Das Bild, das meine Kolleg:innen von mir haben, passt zu dem, was ich jeden Tag leiste.
9. Manchmal fühle ich mich von meinen Kolleg:innen unter Druck gesetzt.
10. Meine Kolleg:innen lassen mich spüren, dass ich viel wert bin.
11. Meine Kolleg:innen zeigen persönliches und fachliches Interesse an mir.
12. Meine Kolleg:innen ermöglichen es mir, Neues zu lernen.
13. Meine Kolleg:innen scheuen keine Kosten oder Mühen, um ein Arbeitsumfeld zu schaffen, in dem ich gut arbeiten kann (Arbeitsumgebung, Materialien, Dekoration und Pflanzen o.ä.).
14. Meine Kolleg:innen teilen auch persönliche und emotionale Themen mit mir.
15. Meine Kolleg:innen geben mir das Gefühl Teil einer Gemeinschaft zu sein.

Trifft nicht zu Trifft zu Kann ich nicht beantworten

**O**	**O**	**O**	**O**	**O**	**O**	**O**	**O**	**O**	**O**	**O**

9.4 Appendix D – The EA-SI Work Scale (Colleagues) Translated for Better Understanding

The following questions relate to the appreciation you experience from others during your workday. Please note that the questions are focusing on how appreciated you feel. There are no right or wrong answers. Please respond honestly and conscientiously. We are interested in your personal feelings and evaluations. Following, there will be statements regarding your colleagues.

Please state to what extent these statements apply to you personally. Think about the last three months. Please choose the option "I cannot answer this" if a question is entirely inapplicable or unanswerable regarding your professional activity.

1. My colleagues take my opinion seriously.
2. My colleagues show me that they know and appreciate my strengths.
3. My colleagues often entrust me with tasks that are important to the entire team.
4. My colleagues repeatedly emphasize that they can rely on me.
5. I feel that my colleagues appreciate my work.
6. I feel seen by my colleagues with all my successes.
7. I receive appropriate material gifts (chocolates, bouquet of flowers, food, small lucky charm, etc.) from my colleagues.
8. The image my colleagues have of me matches what I do every day.
9. Sometimes I feel pressured by my colleagues. (r)
10. My colleagues show me that I am worth a lot.
11. My colleagues show personal and professional interest in me.
12. My colleagues make it possible for me to learn new things.
13. My colleagues spare no expense or effort to create an environment where I can work well (e.g., surroundings, materials, decoration/ plants).
14. My colleagues share personal and emotional issues with me.
15. My colleagues give me the feeling of being part of a community.

I disagree									I agree	I cannot answer this
O	O	O	O	O	O	O	O	O	O	O

9.5 Appendix E – The EA-SI Work Scale (Supervisors)

Die folgenden Fragen beziehen sich auf die Wertschätzung, die Sie innerhalb Ihres Arbeitsalltages durch andere erfahren. Wichtig ist hierbei, dass es darum geht, wie sehr Sie sich persönlich wertgeschätzt fühlen. Es gibt also keine richtigen oder falschen Antworten. Bitte antworten Sie ehrlich und gewissenhaft. Uns interessieren an dieser Stelle Ihre persönlichen Empfindungen und Einschätzungen. Ihnen werden nachfolgend Aussagen präsentiert, die sich auf Ihre direkte Führungskraft beziehen.

Bitte geben Sie an, inwieweit diese Aussagen auf Sie persönlich zutreffen. Erinnern Sie sich hierbei ganz gezielt an die letzten drei Monate. Bitte kreuzen Sie die Option „Kann ich nicht beantworten." nur dann an, wenn diese ganz und gar nicht für Ihre berufliche Tätigkeit zutreffend oder zu beantworten ist.

1. Meine direkte Führungskraft nimmt meine Einwände ernst.
2. Meine direkte Führungskraft zeigt mir, dass sie meine Stärken kennt und schätzt.
3. Meine direkte Führungskraft überträgt mir Verantwortung für wichtige Unternehmensressourcen (Finanzmittel, Personal, Material usw.).
4. Meine direkte Führungskraft betont immer wieder, dass sie sich auf mich verlassen kann.
5. Ich spüre deutlich, dass meine direkte Führungskraft meine Arbeit zu schätzen weiß.
6. Ich fühle mich mit all meinen Erfolgen von meiner direkten Führungskraft gesehen.
7. Ich erhalte angemessene ***materielle*** Geschenke (Bonuszahlung, Wertgutscheine, Pralinen, Präsente o.ä.) von meiner direkten Führungskraft.
8. Das Bild, das meine direkte Führungskraft von mir hat, passt zu dem, was ich jeden Tag leiste.
9. Manchmal fühle ich mich von meiner direkten Führungskraft unter Druck gesetzt.
10. Meine direkte Führungskraft lässt mich spüren, dass ich viel wert bin.
11. Meine direkte Führungskraft zeigt persönliches und fachliches Interesse an mir.
12. Meine direkte Führungskraft lässt mich spüren, dass sie sieht, was ich alles leiste.
13. Meine direkte Führungskraft scheut keine Kosten oder Mühen, um ein Arbeitsumfeld zu schaffen, in dem ich gut arbeiten kann (Arbeitsumgebung, Materialien, technische Ausstattung, benötigte Software, Angebote für gemeinsame Betriebsausflüge/ Aktivitäten o.ä.).
14. Ich kann mit meiner direkten Führungskraft über persönliche und emotionale Themen sprechen.
15. Meine direkte Führungskraft gibt mir das Gefühl Teil einer Gemeinschaft zu sein.

Trifft nicht zu									Trifft zu	Kann ich nicht beantworten
**O**	**O**	**O**	**O**	**O**	**O**	**O**	**O**	**O**	**O**	**O**

9.6 Appendix F – The EA-SI Work Scale (Supervisors) – Translated for Better Understanding

The following questions relate to the appreciation you experience from others during your workday. Please note that the questions are focusing on how appreciated you feel. There are no right or wrong answers. Please respond honestly and conscientiously. We are interested in your personal feelings and evaluations. Following, there will be statements regarding your direct supervisor.

Please state to what extent these statements apply to you personally. Think about the last three months. Please choose the option "I cannot answer this" if a question is entirely inapplicable or unanswerable regarding your professional activity.

1. My direct supervisor takes my opinion seriously.
2. My direct supervisor shows me that they know and appreciate my strengths.
3. My direct supervisor gives me responsibility for important company resources (e.g., financial resources, personnel, materials).
4. My direct supervisor repeatedly emphasizes that they can rely on me.
5. I feel that my direct supervisor appreciates my work.
6. My direct supervisor sees me with all my successes.
7. I receive appropriate material gifts (e.g., bonus payment, vouchers, chocolates, presents) from my direct supervisor.
8. The image my direct supervisor has of me matches what I do every day.
9. Sometimes I feel pressured by my direct supervisor. (r)
10. My direct supervisor shows me I am worth a lot.
11. My direct supervisor shows personal and professional interest in me.
12. My direct supervisor shows me that they see all my efforts.
13. My direct supervisor spares no expense or effort to create an environment where I can work well (e.g., surroundings, materials, technical equipment, required software, company activities).
14. I can talk to my direct supervisor about personal and emotional issues.
15. My direct supervisor gives me the feeling of being part of a community.

I disagree I agree I cannot answer this

**O**	**O**	**O**	**O**	**O**	**O**	**O**	**O**	**O**	**O**	**O**

9.7 Appendix G – Descriptive Measures and Correlation Matrix Study Two

Table G1

Pearson Product-Moment Correlations and Descriptives – Study Two

	M	SD	α	1	2	3	4	5	6	7	8	9	10	11	12	13	14	15	16	17	18
1. EA-SI - Colleagues	7.02	1.52	.90	—																	
2. EA-SI - Supervisors	6.56	1.96	.93	.67**	—																
3. Self-Esteem	4.83	0.81	.89	.26**	.24**	—															
4. Perceived Stress	2.25	0.56	.93	-.36**	-.35**	-.56**	—														
5. AAWS - Colleagues	5.35	1.09	.85	.70**	.52**	.28**	-.27**	—													
6. AAWS - Supervisors	5.02	1.27	.88	.56**	.84**	.25**	-.38**	.54**	—												
7. Appreciation single - C	3.97	0.90	—	.66**	.50**	.23**	-.34**	.60**	.48**	—											
8. Appreciation single - S	5.19	1.63	—	.50**	.79**	.23**	-.41**	.43**	.79**	.51**	—										
9. Interactional Justice - C	4.47	0.68	.70	.37**	.28**	.26**	-.29**	.39**	.34**	.45**	.34**	—									
10. Interactional Justice - S	4.40	0.80	.79	.33**	.50**	.27**	-.33**	.28**	.54**	.37**	.60**	.66**	—								
11. Workplace Ostracism	1.49	0.64	.75	-.27**	-.22**	-.17**	.19**	-.31**	-.28	-.33**	-.21**	-.43	-.34**	—							
12. Attitude Policy	3.53	0.80	.87	-.06	-.05	-.04	.13**	.02	-.05	-.05	-.04	-.03	-.03	.00	—						
13. Emotional Exhaustion	2.81	1.26	.90	-.30**	-.35**	-.41**	.67**	-.27**	-.38**	-.36**	-.41**	-.27**	-.35**	.35**	.05	—					
14. Work Engagement	4.69	1.44	.95	.48**	.49**	.27**	-.37**	.44**	.48**	.42**	.43**	.20**	.23**	-.02	-.05	-.31**	—				
15. Work Satisfaction	4.45	1.58	.84	.53**	.59**	.30**	-.45**	.46**	.55**	.52**	.59**	.31**	.37**	-.13**	-.05	-.46**	.73**	—			
16. Life Satisfaction	5.11	1.07	.87	.29**	.26**	.56**	-.51**	.28**	.29**	.27**	.24**	.21**	.23**	*-.15	-.06	-.38**	.35**	.36**	—		
17. Social Support - C	3.17	0.53	.82	.67**	.45**	.30**	-.35**	.64**	.45**	.58**	.41**	.39**	.31**	-.37**	.02	-.37**	.33**	.42**	.28**	—	
18. Social Support - S	2.99	0.69	.89	.44**	.77**	.27**	-.37**	.42**	.79**	.36**	.74**	.30**	.53**	-.25**	-.04	-.34**	.38**	.49**	.27**	.51**	—

Note. Mean (*M*), standard deviation (*SD*), and Cronbach's alpha (α) are displayed. All correlations were tested one-sided with a total $N = 391$.

* $p<.05$, ** $p<.01$. All analyses are based on T1. Colleagues and supervisors are abbreviated with C and S, environmental policy attitude is abbreviated with attitude policy.

9.8 Appendix H – Misfit Plots of the Confirmatory Factor Analyses in Study Two

Table H1

Misfit Plot of the Confirmatory Factor Analysis in Study Two – Colleagues as Appreciators

	1	2	3	4	5	6	7	8	9	10	11	12	13	14	15
1	**0**														
2	.07	**0**													
3	.05	.05	**0**												
4	.02	.05	.02	**0**											
5	.01	.04	.02	.03	**0**										
6	0	.03	.01	.02	.01	**0**									
7	.02	.05	0	.02	.03	.02	**0**								
8	.02	.02	.04	0	.01	.01	.01	**0**							
9	0	.02	.07	0	.04	.01	.02	.06	**0**						
10	.02	0	.02	.01	.01	.02	.01	.02	.01	**0**					
11	.01	.02	.04	.04	.05	.01	0	.03	.03	.01	**0**				
12	.05	.04	.02	.02	.03	.04	.01	.04	.05	0	.09	**0**			
13	.02	.03	.04	.08	.02	.02	.11	.04	.01	.02	.02	.05	**0**		
14	.01	.01	.04	.04	.04	.02	.04	.03	.06	.03	.03	.07	.01	**0**	
15	0	0	.07	0	.04	.03	.14	.02	.03	.03	.06	0	0	.05	**0**

Note. The items are presented in the order of the EA-SI Work Scale.

Table H2

Misfit Plot of the Confirmatory Factor Analysis in Study Two – Direct Supervisors as Appreciators

	1	2	3	4	5	6	7	8	9	10	11	12	13	14	15
1	**0**														
2	0	**0**													
3	.02	0	**0**												
4	.04	.03	.05	**0**											
5	.03	.01	.03	.01	**0**										
6	.03	0	.02	0	0	**0**									
7	.01	.03	.04	.02	.02	.03	**0**								
8	.01	.01	.10	.06	.01	.01	.10	**0**							
9	.03	.01	.10	.01	.03	.03	.03	.06	**0**						
10	.02	.01	.01	.02	.02	.01	0	.03	.01	**0**					
11	.03	.01	.02	.03	.01	.01	.01	0	.03	.02	**0**				
12	.03	.01	0	.04	.01	.01	0	.01	.02	.01	.01	**0**			
13	.04	.04	.06	.03	.01	.03	.14	.11	.01	.01	.02	.04	**0**		
14	.02	0	.05	.03	.04	.04	.03	0	.06	.01	.10	0	.02	**0**	
15	.04	.04	.01	0	.01	.02	.04	.04	.03	.01	.02	.01	.05	.05	**0**

Note. The items are presented in the order of the EA-SI Work Scale.

9.9 Appendix I – Data Transparency Appendix – Study Two

Variables in the Complete Dataset	Poster (STATUS=presented)	MS 1 (STATUS=current)	MS 2 (STATUS=published)	MS 3 (STATUS=planned)
Experienced Appreciation (Colleagues)		X	X	X
Experienced Appreciation (Supervisors)	X	X	X	X
Perceived Stress	X	X		
Global Self-Esteem	X	X		
Work Satisfaction	X	X		X
Life Satisfaction	X	X		
Work Engagement	X	X		X
Emotional Exhaustion	X	X		X
Appreciation at Work Scale		X		
Appreciation single Item (Colleagues)		X		
Appreciation single Item (Supervisors)		X		
Workplace Ostracism		X		
Interpersonal Justice		X		
Environmental Policy Attitude		X		
Social Support		X		
Big Five Inventory				X
Turnover Intention				
Sleep Quality				
Effort-Reward Imbalance				

Note. The abbreviation „MS“ stands for manuscripts planned or written using Study Two’s data. There is no detailed „Data Transparency Appendix“ on Study One’s data since these data were analyzed in the current manuscript with no further use planned.
